# Are We Jumping to the Wrong Conclusions? Longer Jumps and More Hops in Female Football Players Who Went on to Sustain a Primary or Secondary ACL Injury Compared to Those Who Did Not

**DOI:** 10.1186/s40798-023-00656-7

**Published:** 2023-11-10

**Authors:** Anne Fältström, Joanna Kvist, Martin Hägglund

**Affiliations:** 1 Unit of Physiotherapy, Department of Health, Medicine and Caring Sciences, Linköping University, 581 83 Linköping, Sweden; 2 Region Jönköping County, Rehabilitation Centre, Ryhov County Hospital, 551 85 Jönköping, Sweden; 3 Stockholm Sports Trauma Research Center, FIFA Medical Centre of Excellence, Karolinska Institute, 171 77 Stockholm, Sweden

**Keywords:** Performance, Screening, Soccer

## Abstract

**Background:**

Different functional performance tests are used to assess patients in the clinic and before return to sport (RTS), where the rehabilitation goal is to reach good strength and jumping ability. A limb symmetry index of ≥ 90% is a common target in rehabilitation before RTS. The aim of this short communication is to use data from our 2-year prospective cohort study on female football players, either with or without an anterior cruciate ligament (ACL) reconstruction, to discuss whether hop performance in 3 commonly used hop tests can inform safe football participation, that is, with a low risk for ACL injury or reinjury.

**Method:**

At baseline, 117 active female football players (mean age ± standard deviation, 20 ± 2 years) were included 19 ± 9 months after ACL reconstruction as well as 119 matched female knee-healthy players (age 19 ± 3 years). All players performed a single hop for distance test, 5-jump test and side hop test at baseline and were then prospectively followed for 2 years. Twenty-eight (24%) players sustained a second ACL injury and 8 (7%) sustained a primary ACL injury.

**Results:**

Longer jumps in the 5-jump test (922 cm vs. 865 cm, Cohen’s *d* =  − 0.60) and more hops in the side hop test for both limbs (41–42 hops vs. 33–36 hops, *d* =  − 0.43 to − 0.60) were seen in players who sustained a second ACL injury compared with those who did not. Longer jumps in the single hop for distance test (both limbs) (139–140 cm vs. 124–125 cm, *d* =  − 0.38 to − 0.44), in the 5-jump test (975 cm vs. 903 cm, *d* = −0.42) and more hops in the side hop test (both limbs) (48–49 hops vs. 37–38 hops, *d* =  − 0.38 to − 0.47) were seen in players who sustained a primary ACL injury compared with those who did not.

**Conclusions:**

The average hop performance, i.e. longer jumps or more hops, was greater in players who went on to sustain a primary or secondary ACL injury compared to those who did not over a two-year follow-up period. Even though hop tests are not used in isolation to evaluate readiness to RTS, their interpretation needs consideration in the decision-making process of returning to pivoting sports.

**Supplementary Information:**

The online version contains supplementary material available at 10.1186/s40798-023-00656-7.

## Key Points

• The average hop performance, i.e. longer jumps or more hops, was greater in female football players who went on to sustain a primary or secondary ACL injury compared to those who did not over a two-year follow-up period.

• Even though hop tests are not used in isolation to evaluate readiness to RTS, their interpretation needs consideration in the decision-making process of returning to pivoting sports.

• The relationship between better hop performance and risk of ACL injury is unclear. It is likely not a predictor per se, but better performance could mean more exposure to high risk activities.

**Supplementary Information:**

The online version contains supplementary material available at 10.1186/s40798-023-00656-7.

## Background

Different functional performance tests are commonly used to assess patients in the clinic and before return to sport (RTS) after an anterior cruciate ligament (ACL) injury, where a common rehabilitation goal is to reach good strength and jumping ability, defined as limb symmetry index (LSI) of ≥ 90% [1]. The LSI represents a percentage of the ratio between the injured and the uninjured side. The European Board of Sports Rehabilitation members consider that an LSI ≥ 90% in muscle strength (e.g. in quadriceps and hamstrings) and hop performance in at least 2 hop tests measuring the maximum and endurability hop function is essential for successful RTS, i.e. low risk for new ACL injury and posttraumatic knee osteoarthritis [2]. However, the value of single hop tests to predict new ACL injuries using a cut-off of ≥ 90% LSI is not well elucidated [3–6]. We have previously reported poor ability of commonly used clinical tests (Star excursion balance test, single hop for distance, side hop test, Tuck jump and the drop vertical jump) to predict new ACL injury in female football players with and without ACL reconstruction (ACLR) using proposed LSI cut-offs [3]. In the same cohort of females with ACLR, we found that there was an interaction between functional performance, clinical assessment, and psychological factors and the risk of a reinjury, using classification and regression tree (CART) analysis [7]. Our own [3, 7] and previous research on the clinical value of various hop tests to protect from new or reinjury is inconclusive. The aim of this short communication is to use data from our 2-year prospective cohort study on female football players, either with or without ACLR, to discuss whether hop performance in 3 commonly used hop tests can inform safe football participation, that is, with a low risk for ACL injury or reinjury.

## Methods

This short communication is based on a 2-year prospective cohort study that included 117 active female football players (mean age ± standard deviation [SD], 20 ± 2 years) tested at baseline at a mean 19 ± 9 months (max 39 months) after ACLR, and 119 matched knee-healthy players (age 19 ± 3 years) selected from the same teams as the players with ACLR [8]. Baseline testing was at the beginning of the football season (January to April). All players performed 6 functional performance tests (Star excursion balance test, single hop for distance, 5-jump test, the drop vertical jump, Tuck jump and side hop test) supervised by the same experienced test leader (A.F.). For the present communication, only the results from 3 hop tests, single hop for distance, 5-jump test and side hop test, are used (Table 1). The participants and procedures have been described in detail previously [3, 7, 8].

**Table 1:** Description of the baseline functional performance tests

Baseline tests	Description
Single hop for distance [9] Outcome: maximum length from toe to heel (cm) Evaluates maximum single hop performance	Stand on one leg, jump as far as possible, land on the same leg, with a controlled, balanced landing. Three practice trials and 3 maximum attempts are allowed. If hop length increases in all 3 hops, additional hops are performed until no further increase occurs
5-Jump test [10] Outcome: maximum length from toe to heel (cm) Evaluates lower limb explosive power	Stand on both feet, perform a series of 5 jumps with alternated left and right foot contact, and land with a controlled, balanced landing on both feet. Three practice trials and 3 maximum attempts are allowed
Side hop [9] Outcome: maximal number of side hops where the foot is not touching the tape (*n*) Evaluates hop performance while developing fatigue	Jump on the test leg from side to side outside 2 parallel strips of tape 40 cm apart (with hands behind the back); perform as many jumps as possible for 30 s. If the foot touches the strips of tape, the hop is not valid. A few test hops are performed to get familiarized with the test. One maximum attempt was videotaped and analysed afterwards to count successful jumps

All players reported if they sustained a new ACL injury or not over a 2-year follow-up period (100% response rate). For all reported severe acute knee injuries (seeking medical attention), the diagnosis was checked and confirmed via medical records or from the Swedish National Knee Ligament Register. Of the female players with baseline ACLR, 28 (24%) sustained a new ACL injury (21 ipsilateral and 7 contralateral ruptures), and 8 (7%) of the 119 baseline knee-healthy players sustained a primary ACL injury when playing football [8]. The time frame from testing to new ACL injury was median 8.5 months (range 0–24 months) for the players with ACLR and 12 months (range 0–24 months) for the knee-healthy players. Both self-reported new contact (31%) and non-contact ACL injuries (69%) were included. There were no differences in age, height, weight, playing position, dominant limb (preferred kicking limb), level of play, time after ACLR and follow-up, graft choice, graft diameter, or presence of concomitant injuries at ACLR between players who went on to sustain or did not sustain a new ACL injury [7].

### Statistical Methods

All statistical analyses were performed with SPSS Statistics for Windows (v 27.0; IBM Corp; Armonk, NY). Means ± SD were calculated and also median (interquartile range) for knee-healthy females for descriptive statistics. Between-group comparisons of jump length in the single hop for distance test and the 5-jump test and the number of hops in the side hop test between players who did or did not sustain an ACL injury were made in both groups of players with primary ACLR, using Student’s *t* test, and in knee-healthy players using Mann–Whitney* U* test. Due to potential associations between height and hop performance, all analyses were repeated and adjusted for height (distance and number of hops/height). We also conducted sensitivity analyses specifically on players who went on to sustain a non-contact ACL injury compared to players who did not sustain an ACL injury (for the players with ACLR) during follow-up. Effect sizes with Cohen’s *d* (limits: 0.2, small effect; 0.5, medium effect; 0.8, large effect) were reported. Cohen’s *d* values were transformed from eta square when using Mann–Whitney *U* test. The significance level was set at *P* < 0.05.

## Results

**Fig. 1:**
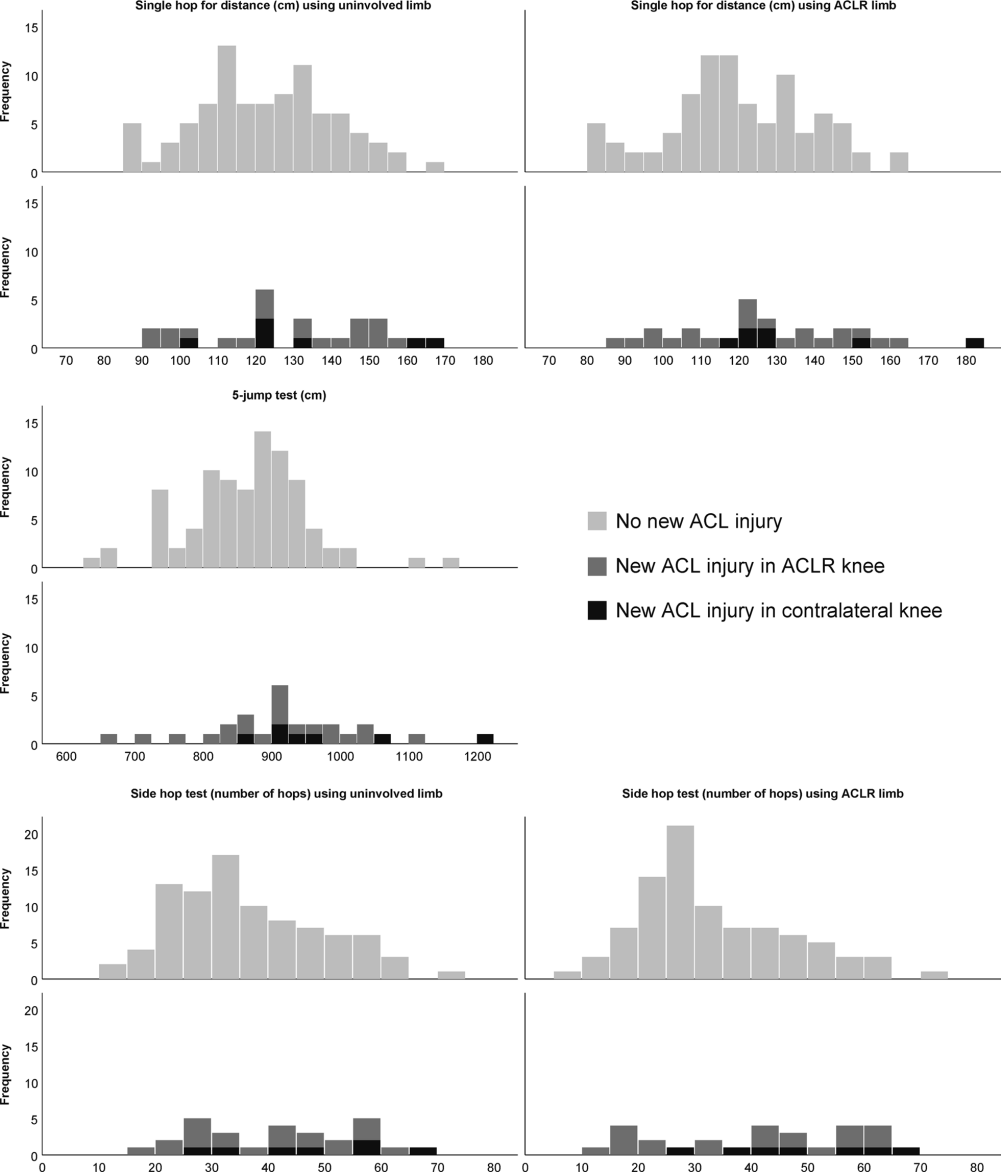
Results for the single hop for distance test, 5-jump test and side hop test in female players with anterior cruciate ligament (ACL) reconstruction (ACLR) who did (*n* = 28) or did not (*n* = 89) sustain a new ACL injury. The uninvolved limb is the non-reconstructed limb and the ACLR limb is the ACL reconstructed limb at baseline. The *x* axis displays hop length in centimetres for the single hop for distance test and the 5-jump test and the number of hops for the side hop test. The *y* axis displays the frequency (numbers) of players

**Fig. 2:**
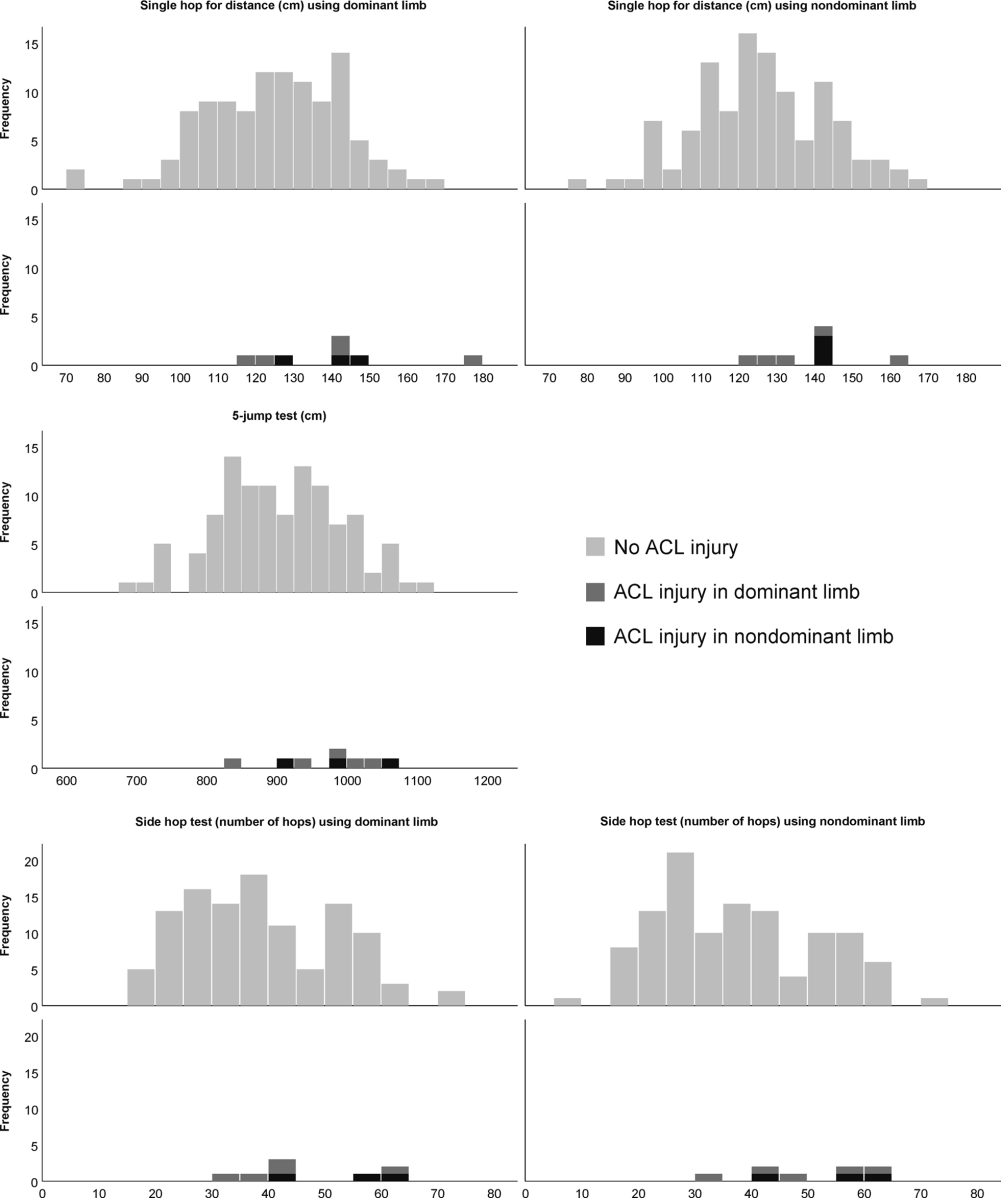
Results for the single hop for distance test, 5-jump test and side hop test in knee-healthy females at baseline who did (*n* = 8) or did not (*n* = 111) sustain an anterior cruciate ligament (ACL) injury. The dominant limb is the preferred kicking leg. The *x* axis displays hop length in centimetres for the single hop for distance test and the 5-jump test and the number of hops for the side hop test. The *y* axis displays the frequency (numbers) of players

There was considerable overlap in jump performance between players who went on to sustain or did not sustain a new ACL injury (Figs. 1, 2). At group level, there was no significant difference in the single hop for distance test in players with ACLR who did or did not sustain a new ACL injury (Table 2, Fig. 1). Longer jumps in 5-jump test (922 cm vs. 865 cm, mean difference (MD) [95% CI]; 57 [16–98] cm, Cohen’s *d =* − 0.60) and more hops in the side hop test for both limbs (41–42 hops vs. 33–36 hops, MD [95% CI]; 6 [0–12] and 9 [2–15] hops, Cohen’s *d =* − 0.43 to − 0.60) were seen in players who sustained a second ACL injury compared with those who did not (Table 2, Fig. 1).

**Table 2:** Hop test results and group differences between players who went on to sustain or did not sustain a new anterior cruciate ligament injury in female football players with (*n* = 117) a previous primary ACL reconstruction

	Females with ACL reconstruction						
	No new ACL injury (*n* = 89)	New ACL injury					
		Rerupture or CACL (*n* = 28)	Rerupture (*n* = 21)	CACL (*n* = 7)	Mean difference^a^ (95% CI)	*P* value^a^	Cohen’s *d*
Single hop for distance, cm							
Uninvolved limb	122 ± 18	128 ± 21	127 ± 21	132 ± 23	− 6 (− 14 to 2)	0.134	− 0.33
ACL reconstructed limb	120 ± 19	127 ± 23	124 ± 22	135 ± 24	− 7 (− 15 to 1)	0.102	− 0.36
Height adjusted (jump length/height)							
Uninvolved limb	0.73 ± 0.11	0.77 ± 0.13	0.74 ± 0.14	0.79 ± 0.11	− 0.04 (− 0.09 to 0.01)	0.093	− 0.37
ACL reconstructed limb	0.71 ± 0.11	0.76 ± 0.13	0.76 ± 0.13	0.80 ± 0.11	− 0.05 (− 0.10 to 0.00)	0.071	− 0.40
5-Jump test, cm							
Both limbs	865 ± 88	922 ± 116	904 ± 113	976 ± 117	− 57 (− 98 to − 16)	**0.007**	− 0.60
Height adjusted (jump length/height)							
Both limbs	5.15 ± 0.52	5.52 ± 0.67	5.41 ± 0.70	5.82 ± 0.47	− 0.37 (− 0.61 to − 0.13)	**0.003**	− 0.66
Side hop, n							
Uninvolved limb	36 ± 13	42 ± 15	39 ± 14	47 ± 15	− 6 (− 12 to − 0)	**0.049**	− 0.43
ACL reconstructed limb^b^	33 ± 13	41 ± 17	39 ± 18	48 ± 13	− 9 (− 15 to − 2)	**0.007**	− 0.60
Height adjusted (number of hops/height)							
Uninvolved limb	0.21 ± 0.08	0.25 ± 0.09	0.24 ± 0.09	0.28 ± 0.08	− 0.04 (− 0.07 to − 0.00)	**0.042**	− 0.45
ACL reconstructed limb	0.20 ± 0.08	0.25 ± 0.10	0.23 ± 0.11	0.29 ± 0.08	− 0.05 (− 0.09 to − 0.01)	**0.006**	− 0.60
Limb symmetry index, %							
Single hop for distance	98 ± 8	99 ± 9	98 ± 10	102 ± 7	− 1 (− 4 to 3)	0.630	− 0.11
Side hop	93 ± 20	98 ± 19	95 ± 19	104 ± 16	− 4 (− 13 to 4)	0.297	− 0.23

Data are means ± standard deviation. Cohen’s *d* with effect size limits: 0.2, small effect; 0.5, medium effect; 0.8, large effect. *P* values in bold type are significant

*ACL* anterior cruciate ligament, *CACL* contralateral rupture

^a^Comparisons between no new ACL injury (*n* = 89) and rerupture or CACL (*n* = 28) using Student’s *t* test

^b^One player did not jump on her ACL reconstructed limb and was therefore not included in the analysis

Longer jumps in the single hop for distance test for both limbs (139–140 cm vs. 124–125 cm, MD [95% CI]; 15 [2–28] and 14 [2–27] cm, Cohen’s *d =* − 0.38 to − 0.44), in the 5-jump test (975 cm vs. 903 cm, MD [95% CI]; 71 [8–135] cm, Cohen’s *d =* − 0.42), and more hops in the side hop test for both limbs (48–49 hops vs. 37–38 hops, MD [95% CI]; 9 [0–19] and 12 [2–22] hops, Cohen’s *d =* − 0.38 to − 0.47) were seen in players who sustained a primary ACL injury compared with those who did not. The results persisted after adjustment for height (Fig. 2, Table 3). There was no difference in LSI between players who went on to sustain a primary or second ACL injury or not (Tables 2, 3). The sensitivity analyses on players who went on to sustain a non-contact ACL injury compared to those who did not sustain an ACL injury (for the players with ACLR) showed the same results as the main analyses including all new (contact and non-contact) ACL injuries (Supplementary material).

**Table 3:** Hop test results and group differences between players who went on to sustain or did not sustain an anterior cruciate ligament injury in female football players (*n* = 119)

	Knee-healthy females at baseline				
	No ACL injury (*n* = 111)	ACL injury (*n* = 8)^a^	Mean difference^b^ (95% CI)	*P* value^b^	Cohen’s *d*
Single hop for distance, cm					
Dominant limb	124 ± 18, 125 (25)	139 ± 18, 141 (23)	− 15 (− 28 to − 2)	**0.040**	− 0.38
Nondominant limb	125 ± 17, 125 (6)	140 ± 13, 141 (7)	− 14 (− 27 to − 2)	**0.019**	− 0.44
Height adjusted (jump length/height)					
Dominant limb	0.74 ± 0.11, 0.76 (0.15)	0.83 ± 0.10, 0.83 (0.12)	− 0.08 (− 0.16 to − 0.01)	0.057	− 0.35
Nondominant limb	0.75 ± 0.10, 0.75 (0.13)	0.83 ± 0.07, 0.83 (0.07)	− 0.08 (− 0.16 to − 0.01)	**0.019**	− 0.44
5-Jump test, cm					
Both limbs	903 ± 89, 902 (125)	975 ± 72, 988 (113)	− 71 (− 135 to − 8)	**0.026**	− 0.42
Height adjusted (jump length/height)					
Both limbs	5.40 ± 0.51, 5.41 (0.67)	5.80 ± 0.38, 5.84 (0.71)	− 0.39 (− 0.76 to − 0.03)	**0.033**	− 0.40
Side hop, n					
Dominant limb	38 ± 13, 38 (23)	48 ± 12, 44 (24)	− 9 (− 19 to 0)	**0.044**	− 0.38
Nondominant limb	37 ± 14, 35 (23)	49 ± 11, 51 (18)	− 12 (− 22 to − 2)	**0.013**	− 0.47
Height adjusted (number of hops/height)					
Dominant limb	0.23 ± 0.08, 0.22 (0.14)	0.28 ± 0.06, 0.26 (0.13)	− 0.05 (− 0.11 to 0.00)	**0.046**	− 0.37
Nondominant limb	0.22 ± 0.08, 0.21 (0.14)	0.29 ± 0.06, 0.30 (0.10)	− 0.07 (− 0.13 to − 0.01)	**0.016**	− 0.45
Limb symmetry index, %					
Single hop for distance	101 ± 8, 100 (9)	101 ± 7, 100 (8)	0 (− 6 to 6)	0.865	0.03
Side hop	98 ± 17, 98 (17)	105 ± 15, 102 (28)	− 7 (− 19 to 5)	0.286	− 0.20

Data are means ± standard deviation and median (interquartile range). Cohen’s *d* values were transformed from eta square with effect size limits: 0.2, small effect; 0.5, medium effect; 0.8, large effect. *P* values in bold type are significant

*ACL* anterior cruciate ligament

^a^Five players injured their dominant limb and 3 players injured their nondominant limb

^b^Comparisons between no ACL injury (*n* = 111) and ACL injury (*n* = 8) using Mann Whitney *U* test

## Discussion

This short communication highlights that the average hop performance, i.e. longer jumps or more hops, was greater in female football players who went on to sustain a primary or secondary ACL injury compared to those who did not over a two-year follow-up period. There was considerable overlap in jump performance between players with new injury/no new injury at the individual level. Even though hop tests are not used in isolation to evaluate readiness to RTS, their interpretation needs consideration in the decision-making process of returning to pivoting sports.

The clinician’s goal is for the player to achieve good strength, e.g. in the quadriceps and hamstrings, and jump ability between limbs to minimize the risk of (re)injury. Our results that better hop performance (longer hop distance and greater number of hops) showed differences between players who sustained or did not sustain a new ACL injury is therefore counterintuitive. The causal relationship is unclear and the clinical implications of our findings need careful consideration. Players with good performance in hop tests probably have better overall function, and psychological readiness to RTS as measured by the ACL-return to sport after injury (ACL-RSI) [11]. Higher ability to generate explosive power may result in higher forces and stress to the knee joint, e.g. in cutting and pivoting movements or landing from a jump, which are common ACL injury mechanisms [12]. Better function could also imply higher self-efficacy, knee-related confidence and more playing time, and thus greater overall football exposure, increasing the risk for ACL injury [13]. This is in line with a recent report that, after ACLR, young athletes with high self-reported knee‐related confidence and who met all RTS strength and hop tests were more likely to sustain a second ACL injury in the first 24 months after RTS [14]. In another report based on ACL registry data, LSI ≥ 90% in quadriceps strength was associated with revision reconstruction, and the authors discussed that patients who achieved a good LSI in quadriceps strength may have returned to sport activities earlier, exposing their knee to graft failure and subsequent revision ACLR [15]. However, our previous analysis, evaluating the same female cohorts as in the current study with the single hop for distance test and the side hop test showed no association between LSI ≥ 90% or LSI with no cutoff and risk of ACL injury [3]. The use of LSI to evaluate injury risk has been questioned because LSI can be overestimated due to poor performance of the uninjured side [1, 2, 16].

**Fig. 3:**
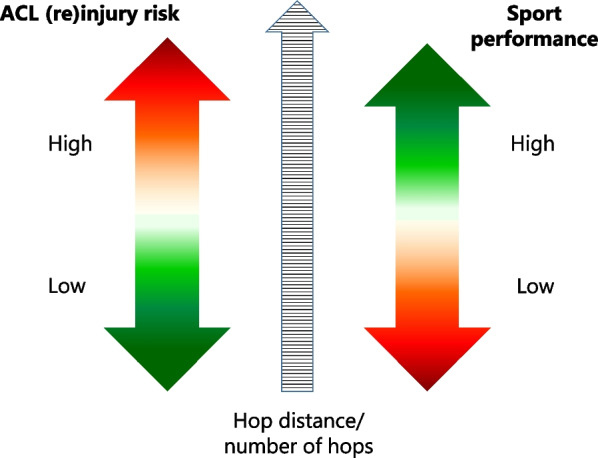
A hypothetical model on how good performance in functional tests allow the athlete to fulfil their goal and return to sport and high sport performance, resulting in high risk for ACL (re)injury

In 1993, Gillquist [17] raised the question if the only effect of ACLR in some individuals is “to give the patients enough security to reach the goal of going back to sports, and then ruining the knee”. According to our results, when symmetrical (LSI values ≥ 90%) and better hop performance was seen in players who went on to incur an ACL injury, we can add to the question whether a good outcome after ACLR followed by rehabilitation, i.e. knee stability, passing muscle strength and functional performance tests criteria (≥ 90% LSI) prior RTS may infer a false security, and increase the risk for a second knee injury when patients RTS (Fig. 3). Participating in sports has many health benefits and not returning to sports affects quality of life [18]. However, the increased risk for injuries has to be taken into account, as some level of risk is unavoidable when playing sports. The question whether the high risk of new ACL injury after ACLR and RTS is acceptable needs careful discussion together with each patient.

The functional testing in the present study was done in the beginning of the football season and not as a RTS test after the ACLR. All tested players had already RTS at the time of testing with different lengths of time after ACLR, which means different exposure to football after the ACLR. These hop tests are commonly used as part of RTS testing after ACLR, but also to evaluate function in e.g. pre-season screening protocols. Isolated potentially modifiable risk factors to sustain a second ACL injury, such as neuromuscular factors (strength, dynamic knee stability, and proprioception) investigated with different functional performance tests, are sparsely reported in the literature [19, 20]. The aetiology of ACL injury is complex and a combination of many factors [21]. We included both self-reported new contact and non-contact ACL injuries in our main analyses, but our sensitivity analysis on players who went on to sustain a non-contact ACL injury specifically (for the players with ACLR) showed the same results. Most of the non-contact and indirect contact ACL injuries in football occur either in a pressing and tackling situation, when players regain balance after kicking, or after landing from a jump, and usually with knee valgus loading [12]. The hop tests reported in the present short communication do not measure specific movement patterns, and in our previous publication [3] the only hop test outcome associated with future ACL injury was having ≥ 6.5 cm knee valgus in the frontal plane measured with the drop vertical jump in knee-healthy players (fair predictive validity). Test batteries with diverse tests that evaluate different functional performance qualities such as strength, hop performance and movement patterns are probably better to support the RTS decision [4–6, 22].

Our findings lead to the question: Should we measure hop performance before RTS? And if we do, what should we evaluate? It is obviously insufficient to only investigate strength and hop performance quantitatively, either in measured performance or using LSI. Other important parameters could be missed. For instance, lower peak knee flexion angle and knee flexion moments during landing in the single hop for distance test have been detected in patients with ACLR despite adequate hop length [23]. The clinician must assess the entire situation and use a multifactorial approach, e.g. body composition, anatomy, age, sex, playing level, sport, psychological and personality factors, surgical factors, and time after ACLR to aid the RTS process. All these factors could be important in protecting against future injury [19, 20]. Many of these factors are proposed as risk factors for ACL reinjury and if the player e.g. is a young female, playing at the elite level in a pivoting sport, has an increased tibial slope, a BMI < 25, has a family history of ACL injury, had an early ACLR after injury, RTS at 6 months post ACLR, and has low psychological readiness to RTS, she would be at high risk for a new ACL injury [19, 20]

## Conclusions

The average hop performance, i.e. longer jumps or more hops, was greater in players who went on to sustain a primary or secondary ACL injury compared to those who did not over a two-year follow-up period. Even though hop tests are not used in isolation to evaluate readiness to RTS, their interpretation needs consideration in the decision-making process of returning to pivoting sports.

### Supplementary Information

**Additional file 1. Table (Supplementary)**. Hop test results and group differences between players who went on to sustain or did not sustain a new non-contact anterior cruciate ligament injury in female football players with (*n* = 117) a previous primary ACL reconstruction

## Data Availability

The datasets used and/or analysed during the current study are available from the corresponding author on reasonable request.

## Abbreviations

ACL: Anterior cruciate ligament
ACLR: Anterior cruciate ligament reconstruction
CACL: Contralateral rupture
LSI: Limb symmetry index
SD: Standard deviation
