# Supplementary material for: Are We Jumping to the Wrong Conclusions? Longer Jumps and More Hops in Female Football Players Who Went on to Sustain a Primary or Secondary ACL Injury Compared to Those Who Did Not
Source: Sports Med Open. 2023 Nov 10;9:105. doi: 10.1186/s40798-023-00656-7 (PMC10638330; doi:10.1186/s40798-023-00656-7)
Supplement: Supplementary file 1 — Additional file 1. Table (Supplementary). Hop test results and group differences between players who went on to sustain or did not sustain a new non-contact anterior cruciate ligament injury in female football players with (n = 117) a previous primary ACL reconstruction [file 40798_2023_656_MOESM1_ESM.docx]

**Supplementary Material. Sport Medicine Open**

Are we Jumping to the Wrong Conclusions? Longer Jumps and More Hops in Female Football Players Who Went on to Sustain a Primary and Second ACL Injury Compared to Those Who Did Not

Anne Fältström^1,2*^, Joanna Kvist^1,3^, Martin Hägglund^1^

^1^Unit of Physiotherapy, Department of Health, Medicine and Caring Sciences, Linköping University, Linköping, 581 83 Sweden

^2^Region Jönköping County, Rehabilitation Centre, Ryhov County Hospital, 551 85 Jönköping, Sweden

^3^Stockholm Sports Trauma Research Center, FIFA Medical Centre of Excellence, Karolinska Institute, 171 77 Stockholm, Sweden

^*^E-mail: [anne.faltstrom@liu.se](mailto:anne.faltstrom@liu.se); [anne.faltstrom@rjl.se](mailto:anne.faltstrom@rjl.se)

|  | Females with ACL reconstruction | | | | |
| --- | --- | --- | --- | --- | --- |
|  | No new ACL injury (*n* = 89) | Non-contact  rerupture or CACL (*n* = 20) | Mean difference^a^  (95% CI) | *P* value^a^ | Cohen’s *d* |
| **Single hop for distance, cm** |  |  |  |  |  |
| Uninvolved limb | 122 ± 18 | 128 ± 21 | −6 (−15 to 3) | 0.211 | −0.31 |
| ACL reconstructed limb | 120 ± 19 | 126 ± 21 | −6 (−16 to 3) | 0.189 | −0.33 |
| Height adjusted (jump length/height) |  |  |  |  |  |
| Uninvolved limb | 0.73 ± 0.11 | 0.77 ± 0.12 | −0.03 (−0.09 to 0.02) | 0.203 | −0.32 |
| ACL reconstructed limb | 0.71 ± 0.11 | 0.75 ± 0.11 | −0.04 (−0.09 to 0.02) | 0.184 | −0.33 |
| **5-jump test, cm** |  |  |  |  |  |
| Both limbs | 865 ± 88 | 910 ± 125 | −45 (−92 to 2) | 0.062 | −0.47 |
| Height adjusted (jump length/height) |  |  |  |  |  |
| Both limbs | 5.15 ± 0.52 | 5.41 ± 0.68 | −0.27 (−0.54 to 0.01) | 0.055 | −0.48 |
| **Side hop, n** |  |  |  |  |  |
| Uninvolved limb | 36 ± 13 | 42 ± 15 | −7 (−13 to 0) | **0.050** | −0.47 |
| ACL reconstructed limb^b^ | 33 ± 13 | 42 ± 17 | −9 (−17 to −0) | **0.012** | −0.73 |
| Height adjusted (number of hops/height) |  |  |  |  |  |
| Uninvolved limb | 0.21 ± 0.08 | 0.25 ± 0.09 | −0.04 (−0.08 to 0.00) | **0.050** | −0.49 |
| ACL reconstructed limb | 0.20 ± 0.08 | 0.25 ± 0.10 | −0.05 (−0.10 to −0.01) | **0.014** | −0.62 |
| **Limb symmetry index, %** |  |  |  |  |  |
| Single hop for distance | 98 ± 8 | 97 ± 20 | −1 (−5 to 3) | 0.691 | −0.10 |
| Side hop | 93 ± 20 | 99 ± 10 | −4 (−13 to 6) | 0.464 | −0.18 |

**Table** (**Supplementary)** Hop test results and group differences between players who went on to sustain or did not sustain a new non-contact anterior cruciate ligament injury in female football players with (*n* = 117) a previous primary ACL reconstruction

Data are means ± standard deviation. Cohen’s *d* with effect size limits: 0.2, small effect; 0.5, medium effect; 0.8, large effect. *P* values in bold type are significant.

ACL, anterior cruciate ligament; CACL, contralateral rupture.

^a^ Comparisons between no new ACL injury (*n* = 89) and non-contact rerupture or CACL (*n* = 20) using Student’s *t* test.

^b^ One player did not jump on her ACL reconstructed limb and was therefore not included in the analysis.
